# Proteomic Landscape of Human Sperm in Patients with Different Spermatogenic Impairments

**DOI:** 10.3390/cells12071017

**Published:** 2023-03-26

**Authors:** Lea Simone Becker, Mohammad A. Al Smadi, Markus Raeschle, Shusruto Rishik, Hashim Abdul-Khaliq, Eckart Meese, Masood Abu-Halima

**Affiliations:** 1 Institute of Human Genetics, Saarland University, 66421 Homburg, Germany; 2 Reproductive Endocrinology and IVF Unit, King Hussein Medical Centre, Amman 11733, Jordan; 3 Department of Molecular Genetics, TU Kaiserslautern, 67653 Kaiserslautern, Germany; 4 Chair for Clinical Bioinformatics, Saarland University, 66123 Saarbrücken, Germany; 5 Department of Pediatric Cardiology, Saarland University Medical Center, 66421 Homburg, Germany

**Keywords:** sperm, male subfertility, oligoasthenozoospermia, asthenozoospermia, proteome

## Abstract

Although the proteome of sperm has been characterized, there is still a lack of high-throughput studies on dysregulated proteins in sperm from subfertile men, with only a few studies on the sperm proteome in asthenozoospermic and oligoasthenozoospermic men. Using liquid chromatography–mass spectrometry (LC-MS/MS) along with bioinformatics analyses, we investigated the proteomic landscape of sperm collected from subfertile men (*n* = 22), i.e., asthenozoospermic men (*n* = 13), oligoasthenozoospermic men (*n* = 9) and normozoospermic controls (*n* = 31). We identified 4412 proteins in human sperm. Out of these, 1336 differentially abundant proteins were identified in 70% of the samples. In subfertile men, 32 proteins showed a lower abundance level and 34 showed a higher abundance level when compared with normozoospermic men. Compared to normozoospermic controls, 95 and 8 proteins showed a lower abundance level, and 86 and 1 proteins showed a higher abundance level in asthenozoospermic and oligoasthenozoospermic men, respectively. Sperm motility and count were negatively correlated with 13 and 35 and positively correlated with 37 and 20 differentially abundant proteins in asthenozoospermic and oligoasthenozoospermic men, respectively. The combination of the proteins APCS, APOE, and FLOT1 discriminates subfertile males from normozoospermic controls with an AUC value of 0.95. Combined APOE and FN1 proteins discriminate asthenozoospermic men form controls with an AUC of 1, and combined RUVBL1 and TFKC oligoasthenozoospermic men with an AUC of 0.93. Using a proteomic approach, we revealed the proteomic landscape of sperm collected from asthenozoospermic or oligoasthenozoospermic men. Identified abundance changes of several specific proteins are likely to impact sperm function leading to subfertility. The data also provide evidence for the usefulness of specific proteins or protein combinations to support future diagnosis of male subfertility.

## 1. Introduction

Infertility defined by the inability to achieve pregnancy within one year of unprotected intercourse is a widespread problem, affecting about 15% of couples worldwide [1]. Infertility can be caused by either male or female reproductive issues. Various medical conditions including malignancies, infections, urogenital conditions, or genetic causes can contribute to male infertility. According to the guidelines of the European Association of Urology (EAU), 30–40% of men in their reproductive age are affected by idiopathic infertility [1]. The routine semen parameters evaluated according to the World Health Organization such as sperm count, motility, and the number of sperm with normal morphology and vitality guidelines are insufficient to fully understand the mechanisms of male subfertility. Towards a better functional understanding, RNAs have been correlated to sperm motility, count, and to a lesser extent to sperm morphology [2,3,4,5]. These studies showed a direct link between the messenger RNAs (mRNAs) and different classes of non-coding RNAs such as microRNAs (miRNAs) in men undergoing infertility treatment. As for the more holistic approaches, a total of 60,505 transcripts have been identified for the sperm transcriptome including 11,688 differentially expressed transcripts in infertile and fertile men (2022) [6]. A total of 6871 proteins have been identified for the sperm proteome, as reviewed by [7]. There is, however, a lack of high-throughput studies on sperm proteome and specifically on dysregulated proteins in sperm of subfertile men. Only few studies focus on the sperm proteome in asthenozoospermic men and no proteomic studies on oligoasthenozoospermic men [8,9,10,11,12,13,14,15,16]. The understanding of male subfertility will largely benefit from a better knowledge of the sperm proteome in general and of the proteins that show differential abundance in specific types of subfertility. Likewise, the function of most sperm proteins awaits further elucidation although first studies on functional clustering did already associated proteins with sperm motility, the capacity of fertilization, structural sperm composition, and sperm energy metabolism [15,17,18]. Here, we employed liquid chromatography mass-spectrometry (LC-MS/MS)-based proteomics to investigate the proteomic landscape and to identified differential abundant proteins of human sperm collected from asthenozoospermic and oligoasthenozoospermic men as compared to normozoospermic controls. Our findings lay the basis to further the discovery of new diagnostic biomarkers for specific forms of male infertility. In the long run, our proteomic data will also help to find therapeutic targets for the treatment of male infertility.

## 2. Materials and Methods

### 2.1. Study Population and Sample Collection

**Figure 1:**
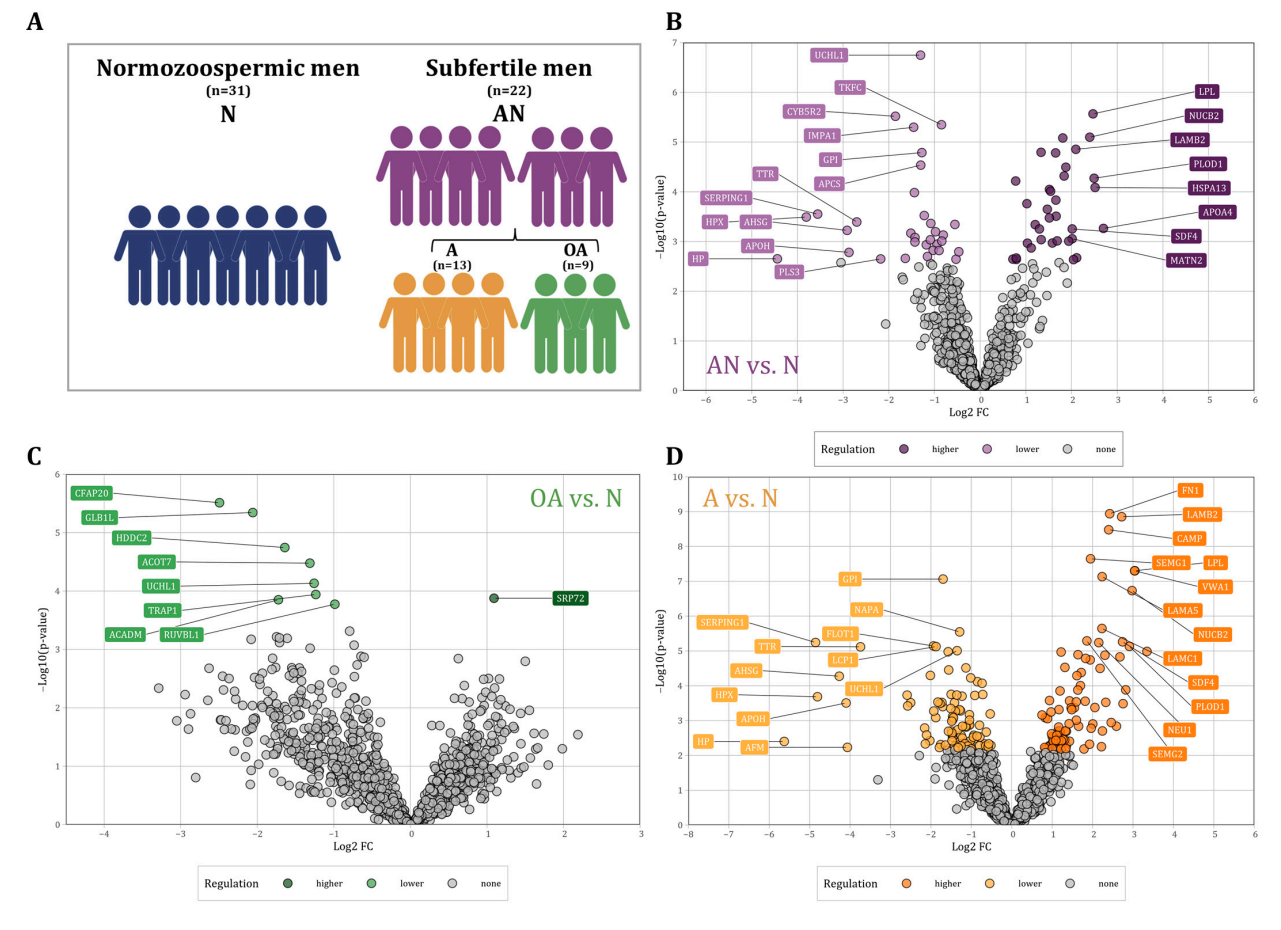
(**A**) Schematic of the study population and their classification into normozoospermic men (N), subfertile men (abnormal, AN) and their specific phenotypes, asthenozoospermic men (A), and oligoasthenozoospermic men (OA). (**B**–**D**): Volcano plot showing the differential abundance levels, i.e., the log2 Fold Change (FC) plotted against the −log10 *p*-value of proteins in sperm collected from (**B**) the subfertile men (*n* = 22) compared to the normozoospermic controls (*n* = 31, AN vs. N), (**C**) the oligoasthenozoospermic men (*n* = 9) compared to the normozoospermic controls (*n* = 31, OA vs. N), and (**D**) the asthenozoospermic men (*n* = 13) compared to the normozoospermic controls (*n* = 31, A vs. N). Significantly abundant proteins were highlighted by color (adjusted *p*-value < 0.05).

Proteomics analysis was performed using sperm samples collected from a total of 53 men attending infertility treatment. Semen samples were classified for primary semen parameters based on WHO 2010 guidelines. These parameters, when taken together, have specified our groups. These groups were specifically classified based on the sperm count (≥15 × 10^6^/mL) and progressive motility (≥32%, motile), resulting in a total of 31 normal men (normozoospermic men, N) and a total of 22 men with at least one abnormal semen parameter (subfertile men, AN). Subfertile men were additionally subdivided into oligoasthenozoospermia (OA, *n* = 9) and asthenozoospermia (A, *n* = 13) as summarized in Figure 1A. More detailed information on the sperm motility of the participants is given in Table S5. All the included men exhibited sperm morphology with an average percentage of ≥4. Semen samples were collected after at least three days of sexual abstinence and immediately liquefied for 30 min at 37 °C. Sperm were purified using discontinuous PureSperm^®^ density gradient (Nidacon international AB, Mölndal, Sweden) to eliminate somatic cells, round cells, and leukocytes as previously described [19,20]. The study complies with the declaration of Helsinki and was approved by the Institutional Review Board (Ha 195/11/updated June 2021) of the Saarland Medical Association. Ethical guidelines were also followed in the conduction of the research, with patients’ and controls’ written informed consent obtained before experiments.

### 2.2. Protein Lysis

Sperm samples were thawed on ice and washed three times with 1× Phosphate-Buffered Saline (PBS) to eliminate contaminants and discontinuous density gradient residues. Samples were mixed with 100 µL of Lysis Buffer [4% SDS, 100 mM Tris/HCl pH 7.6, 0.1 M DTT] and incubated for 5 min at 95 °C. Afterward, each sample was sonicated on ice 10 times each 10 s at 20 joules for 2 s. After 30 min of incubation on ice, samples were centrifuged at 14,000× *g* for 10 min and the supernatant was collected in a separate collection tube and stored at −80 °C.

### 2.3. Peptide Preparation and LC-MS/MS Analysis

To remove SDS from protein lysates, a filter-aided sample preparation (FASP)-method was carried out as described by Wisniewski et al. [21]. Ninety microliters of protein lysate were mixed with 600 µL freshly prepared UA solution [8 M Urea in 0.1 M Tris/HCl pH 8.5], transferred to the Microcon-centrifugal filter unit (MRCF0R030, Merck-Millipore, Darmstadt, Germany) and centrifuged at 14,000× *g* for 15 min at room temperature (RT). Then, the filter unit was washed twice with 200 µL of UA solution at 14,000× *g* for 15 min to remove the remaining SDS. The carbamidomethylation of thiols was achieved with 100 µL of IAA solution (0.05 M iodoacetamide in UA) in a 30-min incubation step in the dark. Residual IAA was eliminated by centrifugation at 14,000× *g* for 15 min at RT and three following washing steps with 100 µL UA solution. After washing the filter units three times with 100 µL of ABC buffer (0.05 M NH4HCO3 in H_2_O), each at 14,000× *g* for 15 min at RT, proteins were digested with 40 µL ABC buffer containing trypsin (trypsin to protein ratio 1:100) overnight in a wet chamber at 37 °C for approximately 18 h. After removing the ABC-trypsin mixture by centrifugation at 14,000× *g* for 15 min, 50 µL of 0.5 M NaCl was added to elute the peptides from the centrifugal filter unit by centrifuging at 14,000× *g* for 15 min at RT. The obtained peptides were acidified with CF3COOH (Trifluoroacetic acid, TFA; Final Conc. 1%) and tested for their acidity with pH paper (pH < 2). Before applying the peptides to the MS-System, digested peptides were cleaned, desalted, and concentrated using the ‘‘Stage Tips technique’’ with self-made C18 filter tips, as described by Rappsilber et al. [22]. After Elution, the peptides were vacuum dried, resuspended in 20 µL of 0.1% Formic Acid (Buffer A) and the concentration of peptides was determined using the Pierce™ Quantitative Colorimetric Peptide Assay kit (Thermo Fisher Scientific Inc., Waltham, Mass., USA). For MS analysis, desalted peptides were resuspended in buffer A (0.1% (*v/v*) formic acid) and separated on a 50 cm reverse phase column with an inner diameter of 75 mm (New Objective from Woburn, MA, USA) packed in-house with 1.8 mm ReproSil-Pur 120 C18-AQ particles (Dr. Maisch GmbH, Ammerbruch-Entrigen, Germany) using a 180 min non-linear gradient of 2–95% buffer B (0.1% (*v/v*) formic acid, 80% (*v/v*) acetonitrile) at a flow rate of 250 nL/min. All MS data were recorded with a data-dependent acquisition strategy. Survey scans were acquired with a resolution of 60,000 at *m/z* = 200. The top 15 most abundant precursors with a charge >2 were selected for fragmentation. MS/MS scans were acquired with a resolution of 15,000 at *m/z* = 200. All other parameters can be obtained from raw files available at the ProteomExchange repository.

### 2.4. Data Processing and Statistical Analysis

The processing of LC-MS/MS data was performed with MaxQuant software (v1.6.3.3, Max Planck Institute of Biochemistry, Martinsried, Germany) [23]. All data were matched with the human reference proteome database (UniProt: UP000005640) with protein and peptide FDR < 1%. The mass spectrometry proteomics data (raw data, MaxQuant Output parameters, and tables) have been deposited to the ProteomeXchange Consortium via the PRIDE [24] partner repository with the dataset identifier PXD039703. Label-free quantified (LFQ) intensities of all protein groups along the 53 samples were preprocessed using R software (v4.2.1, R Core Team, Vienna, Austria). R Script available upon request. Downstream analysis and visualization of results was performed using the R packages operators (v0.1–8), tidyverse (v1.3.2), readxl (v1.4.0), ggplot2 (v3.3.6), VennDiagram (v1.7.3), UpSetR (v1.4.0), pheatmap (v1.0.12), plotly (v4.10.0), gplots (v3.1.3), gridExtra (v2.3), grid (v4.2.1), lattice (v0.20-45), ggrepel (v0.9.1), extrafont (v0.18), showtext (v0.9-5), Rttf2pt1 (v1.3.10), systemfonts (v1.0.4), RColorBrewer (v1.1-3) and/or viridis (v0.6.2). LFQ intensities were log2 transformed and protein groups were filtered to eliminate contaminants, reverse hits, and proteins identified by site only. Protein groups that were identified in at least 70% of all samples were kept for further processing. An unequal variance *t*-test was used to call proteins with significant enrichment in the subfertile men (AN) and their respective subgroups (OA and A) compared to the normozoospermic men (N) and *p*-values were adjusted for multiple testing with the Benjamini–Hochberg procedure [25] and fold changes of the mean LFQ intensities of the respective comparisons of samples were determined. Spearman’s correlation between the LFQ intensities and selected semen parameters (sperm count and progressive sperm motility) was generated using the R package Hmisc (v4.7-2). The correlation coefficients of all proteins are provided in Table S3. To perform ROC and logistic regression analysis, missing values were replaced with values drawn from a normal distribution centered around the detection limit of the MS instrument with a width of 0.3 and a downshift of 1.8 with respect to the standard deviation and mean of all protein intensities of each sample [26]. ROC and logistic regression analysis were performed using the R package pROC (v1.16.0), glmnet (v2.0_18), caret (v6.0_86), broom (v0.5.6), and Matrix (v1.2_18). To increase the predictive potential of a single protein as a biomarker, we build statistical models combining 2 or 3 proteins. Feature selection for a binary logistic regression classification model with L1 regularization was used to reduce the number of proteins tested and obtain the best biomarker combination (LASSO Method). L1 regularization reduces the coefficients of the model features such that some of the features have coefficients of 0, resulting in feature selection. The data were split into a training set (80%) and a test set (20%) for each of the compared classes. The model was fit to the training set and the performance of the model was evaluated against the test set. After selecting features for each of the comparison groups, combinations of size 2 and 3 were selected from the protein subsets, and a logistic regression model was fitted to each of these combinations. Three-fold cross-validation was used to calculate the AUC of the ROC for each of these models to identify the protein combinations that yielded the highest values.

## 3. Results

### 3.1. Basic Semen Characteristics of Patients with Subfertility Compared to Normozoospermic Men

We analyzed sperm samples from normozoospermic (*n* = 31) and subfertile men (*n* = 22) including 9 oligoasthenozoospermic and 13 asthenozoospermic men (Table 1). Normozoospermic men were significantly different in sperm count and progressive motility compared to subfertile men (i.e., oligoasthenozoospermia and asthenozoospermia). Oligoasthenozoospermic and asthenozoospermic men were significantly different in combined sperm count and motility and in combined sperm count, motility, and morphology compared to normozoospermic men (Table 1). Other parameters including age and volume, were not significantly different in these comparisons.

### 3.2. Differentially Abundant Proteins in Sperm as Determined by LC-MS/MS

Proteomic analysis of sperm samples collected from subfertile men (*n* = 22) and normozoospermic men (*n* = 31) was performed using LC-MS/MS analysis. We found a total of 4412 distinct proteins that were present in at least one of the samples. In the following, we restrict our analysis to 1336 proteins that were present in at least 70% of the samples. A total of 204 proteins were differentially abundant in subfertile men as compared to normozoospermic men with a *p*-value < 0.05. (Figure 1). Out of these proteins, 75 proteins showed a higher and 129 a lower abundance level in sperm of subfertile men as compared to normozoospermic men. After adjustment for multiple testing [25], we identified 66 proteins including 34 proteins with significantly higher and 32 proteins with significantly lower abundance levels (Figure 1B). The comparison between oligoasthenozoospermic men (*n* = 9) and normozoospermic men (OA vs. N) revealed 271 differentially abundant proteins with a *p*-value < 0.05 including 203 proteins with lower and 68 proteins with higher abundance levels in sperm of oligoasthenozoospermic men. After adjusting for multiple testing, eight proteins had significantly lower, and one protein had a significantly higher abundance level in oligoasthenozoospermic men (Figure 1C). The comparison between asthenozoospermic men (*n* = 13) and normozoospermic men (A vs. N) identified a total of 417 differentially abundant proteins including 245 proteins with lower and 172 proteins with higher abundance levels in asthenozoospermic men. After adjusting for multiple testing, we identified 181 proteins including 95 proteins with lower and 86 proteins with higher abundance levels in asthenozoospermic men (Figure 1D). These results are summarized in Table S1.

### 3.3. Dysregulated Proteins between Subfertile and Normozoospermic Men

**Figure 2:**
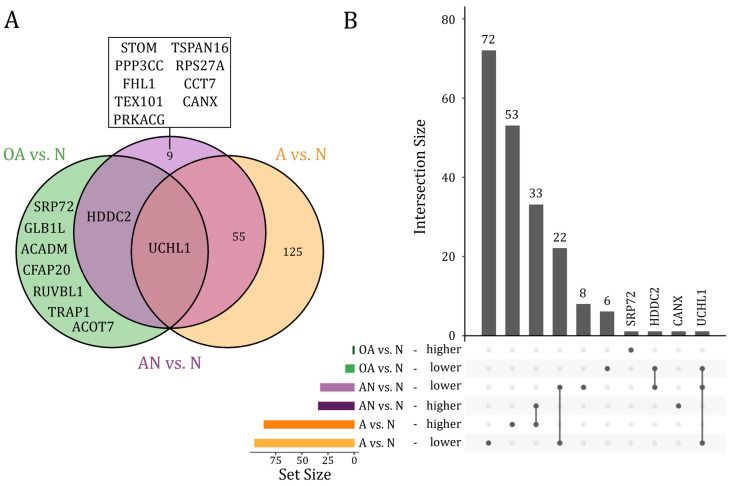
(**A**) Venn diagram and (**B**) Upset plot of proteins that showed significantly different abundance levels (adjusted *p*-value < 0.05) in the comparison of subfertile men compared to the normozoospermic controls (AN vs. N), the oligoasthenozoospermic men compared to the normozoospermic controls (OA vs. N), and the asthenozoospermic men compared to the normozoospermic controls (A vs. N).

We next differentiated between proteins that were dysregulated only in a single, in several, or in all of the above-mentioned comparisons. We restricted these analyses to proteins that were differentially abundant after adjustment for multiple testing (adjusted *p*-value < 0.05). As illustrated in Figure 2, UCHL1 protein was the only protein, which showed a lower abundant level in all comparisons. The HDDC2 protein showed a lower abundant level in both, the specific comparison of OA vs. N and the general comparison AN vs. N. Far more proteins, i.e., 55 proteins were differentially abundant in both, the specific comparison A vs. N and the general comparison AN vs. N, including 22 lower and 33 higher abundant proteins. Likewise, a larger number of proteins (*n* = 125), including 72 lower and 53 higher abundant proteins, were found only in the comparison A vs. N. Few proteins were exclusively found in the comparison OA vs. N (*n* = 7), including six lower abundant proteins (CFAP20, GLB1L, ACADM, ACOT7, TRAP1, RUVBL1) and one (SRP72) higher abundant protein. A total of eight proteins including STOM, PPP3CC, FHL1, TEX101, PRKACG, TSPAN16, RPS27A, and CCT7 were exclusively found with a lower abundant level in the general comparison AN vs. N. CANX was the only protein found in a higher abundant level in the comparison AN vs. N (Figure 2A,B). A detailed list on the proteins is given in Table S2.

### 3.4. Correlation of Proteins with Progressive Sperm Motility and Sperm Count

**Figure 3:**
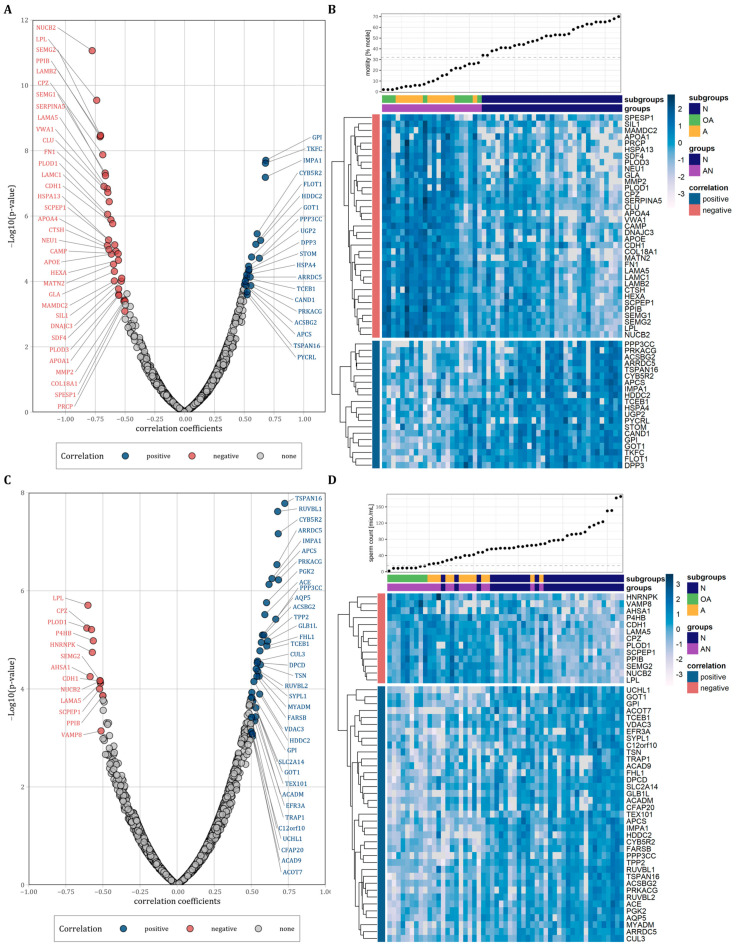
(**A**,**C**): Volcano plots of correlations between protein abundance levels of all identified proteins and sperm motility or sperm count plotted against the −log10 *p*-value, respectively. Correlated proteins were highlighted by color (r > 0.5 and r < −0.5). (**B**,**D**): Heatmaps representing vertically hierarchical clustering of the abundance levels of proteins that were correlated with sperm motility or sperm count, respectively (r > 0.5 and r < −0.5). Horizontally, the proteins were sorted by increasing sperm motility or sperm count, respectively. The colour of rectangles represents z-scored protein abundance levels (white, lower abundant proteins; dark blue, higher abundant proteins; light blue, no change).

We next performed correlation analysis to examine the relationship between protein abundance levels and sperm motility and count. Considering only proteins with a correlation coefficient of r ≤ −0.5 and r ≥ 0.5, we observed 35 proteins that were negatively and 20 proteins that were positively correlated with sperm motility (Figure 3A and Table 2, *p* < 0.05). Figure 3B shows heatmaps for both the positively and negatively correlated proteins with the proteins vertically clustered by their z-score intensity and horizontally sorted by the sperm motility. As expected, the samples of the normozoospermic men showed higher sperm counts and were clearly separated from the samples of the subfertile men. However, the oligoasthenozoospermic and the asthenozoospermic samples were not separated into two different clusters. Using the same correlation coefficients as above, we observed 13 proteins that were negatively and 37 proteins that were positively correlated with sperm count (Figure 3C and Table 2, *p* < 0.05). Figure 3D shows heatmaps for the positively and negatively correlated proteins vertically clustered by their z-score intensity and horizontally sorted by the sperm motility. In contrast to sperm count, the samples of normozoospermic and subfertile men were not clearly separated by sperm motility with normozoospermic samples interspersed within the cluster of the samples from subfertile men and vice versa subfertile samples interspersed within the cluster of normozoospermic samples. The results of the correlation analyses are summarized in Table S3.

### 3.5. Directions of Deregulation and Evidence for Diagnostic Accuracy

**Figure 4:**
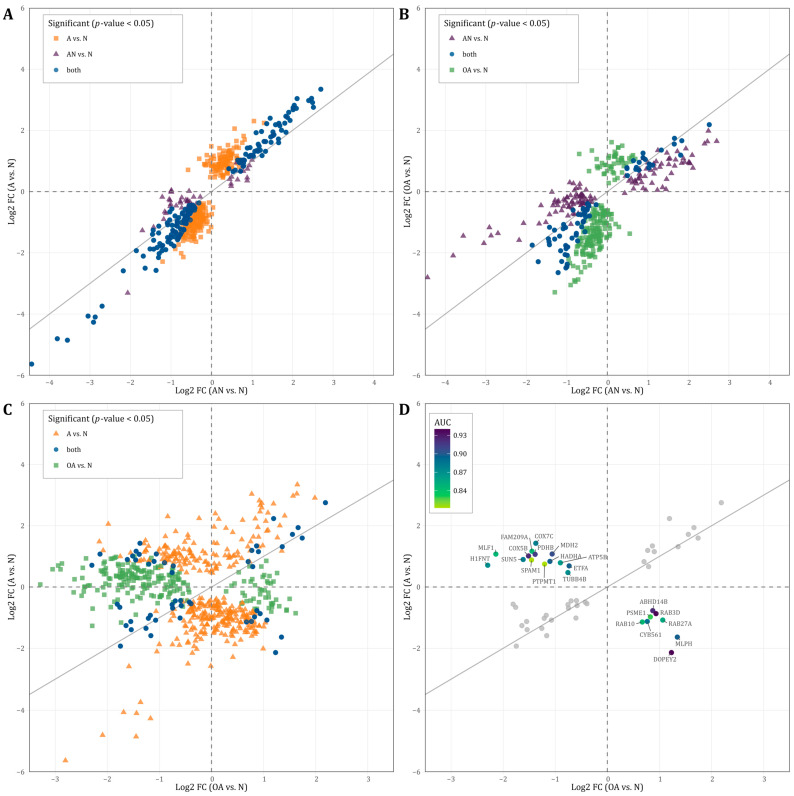
(**A**–**C**): Scatterplot displaying the direction of regulation (log2 fold change) of proteins that showed significantly different abundance levels (*p*-value < 0.05) in the (**A**): AN vs. N and/or OA vs. N, (**B**): AN vs. N and/or A vs. N, and (**C**): A vs. N and/or OA vs. N. (**D**): Scatterplot displaying the direction of regulation (log2 fold change) of proteins that showed significantly different abundance levels (*p*-value < 0.05) in both A vs. N and OA vs. N. The color represents the area under the curve (AUC) value of proteins that were oppositely regulated.

Considering the significant dysregulated proteins listed in Table S1 (*p* < 0.05), we further analyzed the identified proteins based on the direction of regulation of the general (i.e., AN vs. N) and the specific comparisons (OA vs. N, and A vs. N). As shown in Figure 4A, most proteins that were upregulated in AN vs. N were also upregulated in OA vs. N. Likewise, most proteins downregulated in AN vs. N were also regulated in the same direction in OA vs. N. Several proteins showed the same fold change of deregulation in both comparisons. Similarly in Figure 4B, most proteins that were upregulated in AN vs. N were also upregulated in A vs. N. Likewise, most proteins downregulated in AN vs. N were also regulated in the same direction in A vs. N. Several proteins showed the same fold change of deregulation in both comparisons. The correlation between the two comparisons A vs. N and AN vs. N was even more pronounced. Here, we found an extended number of proteins that were deregulated with the same fold change in both comparisons. Several commonly deregulated proteins were strongly downregulated with a fold change below −3. The comparison of proteins, which are significantly deregulated in both or one of the specific comparisons A vs. N and/or OA vs. N, reveals contradictory findings (Figure 4C). Many proteins are inversely regulated depending on the comparison. A total of 14 proteins (FAM209A, COX7C, MLF1, COX5B, PDHB, MDH2, H1FNT, SUN5, HADHA, SPAM1, ATP5B, ETFA, PTPMT1, TUBB4B), which are significant in both comparisons are lower in their abundance level in the OA vs. N comparison and higher in their abundance level in the A vs. N comparison (Figure 4C). A total of eight proteins (ABHD14B, PSME1, RAB3D, RAB10, RAB27A, CYB561, MLPH, DOPEY2) showed a higher abundance level in OA vs. N comparison and a lower abundance level in A vs. N comparison (Figure 4C).

To explore the diagnostic value of the identified proteins, which are shown in Figure 4C, we tested these proteins for their predictive value to discriminate between the different subgroups. As shown in Figure 4D, the AUC values of these proteins ranged between 0.81–0.94 indicating that these proteins can be used to distinguish men with oligoasthenozoospermia and asthenozoospermia (Figure 4D).

### 3.6. Prediction of Subfertility and Their Specific Phenotypes Oligoasthenozoospermia and Asthenozoospermia

**Figure 5:**
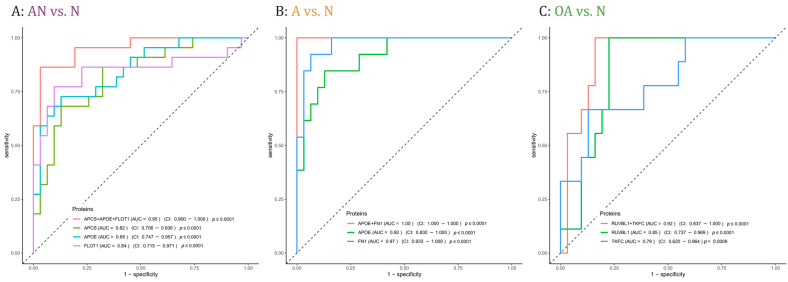
Receiver operating characteristic (ROC) curves comparing sensitivity and specificity of single proteins and protein combinations in predicting (**A**): subfertility (AN), (**B**): asthenozoospermia (**A**,**C**): oligoasthenozoospermia (OA) as compared to normozoospermic controls (N). Area under the curve (AUC) values, the confidence interval (CI), and the *p*-values of single proteins and protein combinations were indicated.

Proteins with adjusted significant *p*-values in at least one comparison were cross-matched with the proteins that were correlated with either sperm count and/or motility (i.e., r ≤ −0.5 or r ≥ 0.5, *p*-value < 0.05). The crossmatch yielded 62 proteins. To determine the predictive potential value of these proteins, we employed feature selection along with 3-fold cross-validation (CV). Table S4 lists the AUC values of single proteins and combinations of 2 or 3 proteins, the mean CV AUC, the confidence interval, and the *p*-values for each comparison, i.e., AN vs. N, A vs. N, and OA vs. N. The combination of the three proteins APCS, APOE, and FLOT1 showed the best AUC value to discriminate subfertile from normozoospermic controls with a mean AUC of 0.95 (Figure 5A). In addition, 5 combinations of two or three proteins showed a perfect AUC value to predict men with asthenozoospermia in the A vs. N comparison with a mean AUC of 1 including the combination of the two proteins APOE and FN1 with a mean AUC of 1 (Figure 5B). As for the discrimination between men with oligoasthenozoospermia and normozoospermic men, the combination of RUVBL1 and TKFC proteins showed the best AUC value with a mean AUC of 0.93 (Figure 5C).

## 4. Discussion

Large-scale MS-based proteomics was used for a comprehensive view on the proteomic landscape of human sperm collected from asthenozoospermic and oligoasthenozoospermic men. In total, we identified 1336 proteins that were present in at least 70% of all sperm samples. The comparison between subfertile men and controls (AN vs. N) showed 66 dysregulated proteins, the comparison between asthenozoospermic with the normozoospermic men (A vs. N) showed 181 proteins, and the comparison between oligoasthenozoospermic and normozoospermic men (OA vs. N) revealed nine proteins.

Notably, the sum of the results obtained from asthenozoospermic and oligoasthenozoospermic men compared to normozoospermic controls (A vs. N and OA vs. N) is not equivalent to the results obtained from subfertile men and controls (AN vs. N). In detail, nine proteins were exclusively identified by comparing abnormal and normal men (Figure 2A). Most of these proteins, namely STOM, PPP3CC, FHL1, TEX101, PRKACG, and TSPAN16, are correlated with sperm motility and/or count (Figure 3/Table 2). Some of the above-mentioned proteins have previously been found to be associated with infertility. Testis-expressed protein 101 (TEX101) is well-studied glycoprotein and highly related to male fertility (as reviewed in [27]). Knockout models of TEX101 and its counterpart protein contributes to the maintenance of spermatogenesis and production of fertile sperm [28]. Similarly, Serine/threonine-protein phosphatase 2B catalytic subunit gamma isoform (PPP3CC) is a catalytic subunit of the sperm-specific isoform of calcineurin and is highly related to male fertility [29]. Specifically, the knockout of PPP3CC in mice reduced the sperm motility and leads to male infertility [29]. Protein Kinase cAMP-Activated Catalytic Subunit Gamma (PRKACG) is a catalytic subunit of protein kinase A (PKA), which is involved in energy metabolism, hyperactivation, and capacitation of sperm [30,31].

The proteins found in our study are largely consistent with the proteins identified in sperm of unaffected men (as reviewed by [7]). We, however, identified 70 additional proteins including nine proteins, namely ESPN (B1AK53), MATN2 (O00339), SERPING1 (P05155), NEU1 (Q99519), GLIPR2 (Q9H4G4), ANKFY1(Q9P2R3), GPR64 (Q8IZP9-9), MYO1C (O00159-2), CABYR (iso3) (O75952-3, O75952-5, O75952-4, G9BQT7) that were dysregulated in at least one of our comparisons. According to the Human Protein Atlas (proteinatlas.org) [32], these proteins are also expressed in the developing germ cells and/or in the testicular somatic cells, i.e., Sertoli cells. We would like to point out that our stringent inclusion criteria for the identified proteins and the relation to testicular cells and/or tissues provided by the Human Protein Atlas strongly suggest that these proteins essentially contribute to the sperm proteome. Additionally, the crossmatch between the proteins identified in this study and the proteins, which were identified by others in asthenozoospermic men [8,9,14,16] yielded at least 10 matched proteins. Of these identified proteins, four proteins, namely ELSPB1, ECH1, GK2, and HSPA9, showed contradictory directions of regulation and six proteins, namely SEMG1, IMPA1, PARK7, LTF, SDHA, and CLU showed the same direction of regulation for asthenozoospermic men. These findings underline the complexity of proteomic regulations in sperm: the small overlap indicates that further studies are likely to identify additional deregulated proteins and the differences in the regulation direction suggest that the direction of regulation depends on further variables such as the biological context.

Sperm do not only deliver paternal DNA but also RNA and proteins to the maternal oocyte [7,33,34]. Our analysis of the sperm proteome contributes to deepen our understanding of the functions and the networks of sperm proteins. Some of the identified proteins are known to play a role specifically in the maintenance of physiological sperm functions, such as energy metabolism and in sperm tail structural composition and mechanics. While most of the proteins were dysregulated in the same direction in all comparisons, we also found proteins that were inversely regulated in specific comparisons such as in asthenozoospermic vs. oligoasthenozoospermic samples (Figure 4D). The proteins PDHB, ATP5B, COX7C, COX5B, MDH2, ETFA, and HADHA all of which showed a lower abundance level in oligoasthenozoospermic men and a higher abundance level in asthenozoospermic men, were involved in metabolism (HSA-1430728) [35] and partly in the generation of precursor metabolites and energy (GO:0006091) [36,37]. The proteins RAB10, RAB3D, RAB27A, and MLPH that showed a lower abundance level in asthenozoospermia and a higher abundance level in oligoasthenozoospermia were either Ras-related or were associated with sperm motility and capacitation [38,39].

Out of the 1336 proteins 55 proteins were correlated with sperm motility including 35 negatively and 20 positively correlated proteins. Likewise, 50 proteins were correlated with sperm count including 13 negatively and 37 positively correlated proteins (Figure 3). The hierarchical clustering of the proteins negatively correlated with motility indicates a cluster within the subfertile men (Figure 3B). This subcluster is composed of samples with a motility below 32%. Notably, the decline in male fertility potential is globally discussed and the semen parameters contributing to male fertility have been declined in recent decades [40]. The sperm count decreased by 50–60% between 1973 and 2011 [41] and sperm motility diminished by 10% between 2002 and 2017 [42]. In the future such proteomic analysis may help to define a new lower limit for sperm motility and may improve the subgrouping of subfertile men.

Current diagnostic methods including physical examination, endocrine, genetic and biochemical testing and semen analysis fail in 30–40% of infertility cases [43]. Protein-based biomarkers may help to characterize and to better understand the biological causes of these cases of idiopathic male infertility. We identified three sets of proteins that may serve as potential biomarkers to predict subfertility and specifically asthenozoospermia and oligoasthenozoospermia (Figure 5). In detail, the combination of the three proteins APCS, APOE, and FLOT1 predicts male subfertility in general, the two combined proteins APOE and FN1 predicts asthenozoospermia, and the two combined proteins RUVBL1 and TFKC oligoasthenozoospermia. The power and robustness of these prediction need of course to be validated by an independent and extended set of samples. As well as their potential as biomarkers, APCS, APOE, FLOT1, FN1, and RUVBL1 proteins have been recognized to play a crucial function in the development of male subfertility. By contrast, the TFKC protein has not yet been related to defined biological processes in sperm production or sperm function. Our findings show a lower abundance level of serum amyloid P-component (APCS) in sperm of asthenozoospermic men, and a positive correlation between APCS and sperm motility and sperm count. These findings are in agreement with previous studies reporting a correlation between APCS and sperm motility and count in seminal plasma. Previous studies also suggested that APCS may be used as a biomarker for low sperm concentrations [44]. APCS is localized on the surface of mature sperm specifically in the tail of spermatozoa and plays a physiological role in reproduction, more precisely in tail-associated functions such as sperm motility [45]. APCS protein was identified in sperm, seminal plasma, and testicular tissues collected from the male reproductive tract [45]. With no APCS-deficiency reported for humans, the role of this protein in human reproduction awaits further evaluation. Likewise, we found a lower abundance level of flotillin-1(FLOT1) in asthenozoospermic men and a positive correlation of FLOT1 with sperm motility. FLOT1 is a scaffolding protein of the tyrosine kinase family, which is involved in the capacitation and acrosome reaction of sperm [46]. As shown for the porcine and mouse sperm acrosome, FLOT1 that is organized in lipid rafts changes its dispersed pattern to an accumulation at the apical ridge of the acrosome during capacitation [46,47,48]. However, the exact function of FLOT1 in sperm and their potential involvement in sperm motility is still not well understood and needs further elucidation. The apolipoprotein E (APOE) was also found in higher abundance levels in asthenozoospermic men and was negatively correlated with sperm motility. The impact of APOE on male infertility was extensively studied on different genotypes and its potential impact on steroidogenesis has been investigated [49,50]. Specifically, the lower fertility potential of specific genotypes accompanied by the decrease in APOE concentration suggests an association with male infertility [49,50]. Thus, it has been suggested that the APOE is involved in sperm maturation during epidydimal transit [51]. Very recently, Liu et al. observed a markedly lower total and progressive motility sperm in ApoE-knockout mice compared to wild type control mice and concluded that ApoE knockout effect male reproductive capacity [52]. In addition to the protein abundance levels, post-translational modifications such as APOE glycosylation likely influence the sperm function thereby complicating the search of suitable biomarkers of male fertility [49,53,54]. Additionally, the importance of APOE Receptor 2 (apoER2) for sperm development was previously demonstrated [55]. The apoER2 protein was expressed in the epididymis and its knockout leads to male infertility in mice [55]. Our results showed that sperm-derived Fibronectin 1 (FN1) was negatively correlated with sperm motility and showed a higher abundance level in men with asthenozoospermia. In agreement with our findings, Wennemuth et al. (1997) observed a negative correlation between sperm motility and seminal fibronectin concentration in men with oligoasthenoteratozoospermia [56]. The glycoprotein FN that is expressed on the sperm surface plays a role in sperm-oocyte interaction and fertilization, and was used as a biomarker for selection in assisted reproductive medicine [57]. Notably, FN binds to sperm-specific integrins that can trigger intracellular signaling pathways. The increase in intracellular Ca^2+^ in turn triggers the cyclic adenosine monophosphate/protein kinase A pathway (cAMP/PKA) and enables the sperm capacitation and ultimately the fertilization [58,59]. Hyperactivation of sperm during sperm capacitation alters the movement pattern and bending of the sperm flagellum. Since transcription is silenced in sperm, hyperactivated sperm motility is achieved through post-translational modification, primarily phosphorylation. PKA can phosphorylate AMP-activated protein kinase (AMPK), which regulates sperm motility in a Ca^2+^-dependent manner [31]. Calle-Guisado et al. (2017) have observed that activation of AMPK beyond physiological levels results in a reduction in sperm motility [60]. We suggest that the higher abundance level of FN1 in asthenozoospermic men could lead to an overactivation of the cAMP/PKA pathway and ultimately to a reduction in sperm motility. 

Our data also show that the RuvB Like AAA ATPase 1 (RUVBL1) protein has a lower abundance level in oligoasthenozoospermic men and is only positively correlated with sperm count. RUVBL1 functions as a DNA-dependent ATPase with ATP-dependent DNA helicase activity and is involved in several transcription complexes and histone modifications [61,62,63]. RUVBL1 interacts with the testis/sperm-specific Small Kinetochore-Associated Protein (SKAP1) and is located in the sperm flagellum [64]. In addition, RUVBL1 is a part of the R2TP complex, which acts as a co-chaperon together with the heat shock protein 90 (HSP90) [65]. HSP90 is also located in the sperm flagellum and is crucial to maintain male fertility. It was hypothesized that a co-chaperon complex is formed in sperm thereby contributing to the maturation of sperm [64,65]. The RUVBL1 protein was positively correlated with sperm count. The sperm count reduction can be caused by environmental factors, testis-related diseases, infections, and obstructions of the male reproductive system [66,67,68,69]. However, cellular mechanisms that reduced the number of sperm in the ejaculate are not well understood. We speculate that defects, lower levels, or even the absence of structural proteins that contribute to the ciliary axoneme or flagellum might stimulate the apoptotic machinery or disrupt spermatogenesis, ultimately leading to a decrease in sperm count in men.

As well as their potential as diagnostic biomarkers, several identified proteins might be used as new therapeutic targets. In agreement with others, our study suggests that the lower or higher abundance of specific proteins impact male fertility. Some of the above-mentioned proteins (APOE, TEX101, PPP3CC) were previously functionally validated in knockout-studies of mice. The absence of these proteins leads to impaired spermatogenesis and/or reduced motility and ultimately to infertility. Current studies are investigating whether such proteins are suitable for the treatment of infertility. For example, Kim et al. developed a nanoparticle complex with PIN1 proteins inside to treat infertile mice that were lacking the PIN1 protein. These experiments successfully restore the fertility of the mice [70]. It has recently been suggested that proteins offer advantages over other small molecules regarding their use as therapeutic agents. They are highly specific and bear less risks of adverse effects as compared to gene therapies [70,71].

## 5. Conclusions

In conclusion, we report the proteomic landscape of human sperm collected from asthenozoospermic and oligoasthenozoospermic men as compared to normozoospermic controls. We identified numerous proteins with differential abundance levels between subfertile and normozoospermic men, between asthenozoospermic and normozoospermic men, and between oligoasthenozoospermic and normozoospermic men. Furthermore, we found numerous proteins correlated with sperm motility and sperm count. Several of these proteins were involved in sperm lipid composition and remodelling, the extracellular matrix, the glycocalyx, the ciliary movement, and/or energy metabolism. There are also several proteins with known involvement in sperm-specialized functions, such as sperm capacitation and fertilization. We identified protein combinations of potential diagnostic value for subfertility in general and asthenozoospermia and oligoasthenozoospermia, specifically. As well as their diagnostic potential, these proteins may offer themselves as possible new therapeutic targets.

## Figures and Tables

**Table 1:** Basic semen characteristics of the comparisons subfertile vs. normozoospermic controls (AN vs. N), oligoasthenozoospermic men vs. normozoospermic controls (OA vs. N) and asthenozoospermic men vs. normozoospermic controls (A vs. N).

Parameters	N (*n* = 31)	AN (*n* = 22)	OA (*n* = 9)	A (*n* = 13)	N vs. AN	N vs. OA	N vs. A
Age	35.8 ± 7.3	33.5 ± 8.3	33.2 ± 7.2	33.7 ± 9.3	n.s.	n.s.	n.s.
Volume (mL)	3.3 ± 1.8	4.1 ± 2.8	4.5 ± 3.4	3.8 ± 2.4	n.s.	n.s.	n.s.
Count (10^6^/mL)	86.7 ± 39.6	26.9 ± 19.6	9.1 ± 3.4	39.2 ± 16.3	***	***	***
Motility (% motile)	52.0 ± 10.6	12.3 ± 9.0	14.9 ± 11.3	10.5 ± 7.0	***	***	***
Morphology (%)	7.0 ± 8.2	9.6 ± 5.7	6.2 ± 3.8	11.9 ± 5.8	n.s.	n.s.	*

Data were presented as mean ± standard deviation. Unpaired two-tailed *t*-test was performed. *p*-value < 0.05 was considered as statistically significant. *** *p* < 0.001; * *p* < 0.05; n.s. = not significant.

**Table 2:** Correlation of protein abundance level as determined by LC-MS/MS with the basic semen parameters sperm motility and sperm count.

**A: Negatively Correlated Proteins**							
**Experimental ID**	**Gene Symbol**	**Motility (% motile)**			**Count (10^6^/mL)**		
		**r**	***p*** **-Value**	**Correlation**	**r**	***p*** **-Value**	**Correlation**
3707	NUCB2	−0.78	8.65 × 10^−12^	negative	−0.52	6.90 × 10^−5^	negative
2530	LPL	−0.74	2.86 × 10^−10^	negative	−0.6	1.97 × 10^−6^	negative
3510	LAMB2	−0.71	3.75 × 10^−9^	negative	−0.5	1.70 × 10^−4^	-
2938	PPIB	−0.71	3.53 × 10^−9^	negative	−0.5	1.37 × 10^−4^	negative
3774	SEMG2	−0.71	3.38 × 10^−9^	negative	−0.52	6.75 × 10^−5^	negative
4530	CPZ	−0.69	1.33 × 10^−8^	negative	−0.58	6.18 × 10^−6^	negative
4609	VWA1	−0.68	1.24 × 10^−7^	negative	−0.42	3.04 × 10^−3^	-
2475	SEMG1	−0.67	4.83 × 10^−8^	negative	−0.49	1.84 × 10^−4^	-
2497	SERPINA5	−0.66	5.81 × 10^−8^	negative	−0.5	1.38 × 10^−4^	-
2521	APOA4	−0.65	7.96 × 10^−6^	negative	−0.39	1.50 × 10^−2^	-
1961	LAMA5	−0.65	1.45 × 10^−7^	negative	−0.52	7.64 × 10^−5^	negative
3782	PLOD1	−0.65	8.84 × 10^−7^	negative	−0.61	5.77 × 10^−6^	negative
2673	CLU	−0.64	1.89 × 10^−7^	negative	−0.4	3.29 × 10^−3^	-
3308	HSPA13	−0.64	5.39 × 10^−6^	negative	−0.49	1.00 × 10^−3^	-
5763	NEU1	−0.64	1.07 × 10^−5^	negative	−0.5	1.04 × 10^−3^	-
2431	FN1	−0.63	3.64 × 10^−7^	negative	−0.46	4.95 × 10^−4^	-
2420	APOE	−0.62	1.47 × 10^−5^	negative	−0.33	3.44 × 10^−2^	-
2675	LAMC1	−0.62	1.30 × 10^−6^	negative	−0.42	1.99 × 10^−3^	-
2722	CDH1	−0.6	1.71 × 10^−6^	negative	−0.52	6.87 × 10^−5^	negative
1832	MATN2	−0.6	2.98 × 10^−5^	negative	−0.33	3.12 × 10^−2^	-
2516	GLA	−0.59	4.95 × 10^−5^	negative	−0.37	1.60 × 10^−2^	-
4767	MAMDC2	−0.59	9.70 × 10^−5^	negative	−0.45	4.17 × 10^−3^	-
6191	SCPEP1	−0.59	7.62 × 10^−6^	negative	−0.52	9.98 × 10^−5^	negative
131	CTSH	−0.58	1.19 × 10^−5^	negative	−0.47	4.93 × 10^−4^	-
1642	CAMP	−0.56	1.42 × 10^−5^	negative	−0.28	4.04 × 10^−2^	-
2531	HEXA	−0.56	2.26 × 10^−5^	negative	−0.47	5.30 × 10^−4^	-
2419	APOA1	−0.55	2.68 × 10^−4^	negative	−0.32	4.91 × 10^−2^	-
2096	PLOD3	−0.55	2.57 × 10^−4^	negative	−0.42	7.00 × 10^−3^	-
5836	SDF4	−0.55	1.70 × 10^−4^	negative	−0.26	1.06 × 10^−1^	-
3918	DNAJC3	−0.53	9.86 × 10^−5^	negative	−0.41	3.37 × 10^−3^	-
6045	SIL1	−0.53	8.01 × 10^−5^	negative	−0.46	8.97 × 10^−4^	-
3180	COL18A1	−0.51	4.09 × 10^−4^	negative	−0.32	3.42 × 10^−2^	-
2572	MMP2	−0.51	3.76 × 10^−4^	negative	−0.26	8.85 × 10^−2^	-
3228	PRCP	−0.5	8.23 × 10^−4^	negative	−0.43	5.04 × 10^−3^	-
4643	SPESP1	−0.5	4.02 × 10^−4^	negative	−0.11	4.58 × 10^−1^	-
2536	P4HB	−0.48	2.41 × 10^−4^	-	−0.56	1.05 × 10^−5^	negative
5898	VAMP8	−0.4	1.04 × 10^−2^	-	−0.51	7.20 × 10^−4^	negative
3601	HNRNPK	−0.38	7.34 × 10^−3^	-	−0.57	1.81 × 10^−5^	negative
2304	AHSA1	−0.35	2.47 × 10^−2^	-	−0.59	5.60 × 10^−5^	negative
**B: positively correlated proteins**							
**Experimental ID**	**Gene symbol**	**Progressive sperm motility**			**Sperm count**		
		**r**	***p*** **-value**	**Correlation**	**r**	***p*** **-value**	**Correlation**
4245	TKFC	0.68	2.41 × 10^−8^	positive	0.49	1.83 × 10^−4^	-
2526	GPI	0.68	1.97 × 10^−8^	positive	0.5	1.18 × 10^−4^	positive
3026	IMPA1	0.68	6.53 × 10^−8^	positive	0.64	5.63 × 10^−7^	positive
4778	HDDC2	0.64	5.55 × 10^−6^	positive	0.56	1.27 × 10^−4^	positive
3299	PPP3CC	0.63	1.96 × 10^−5^	positive	0.67	3.80 × 10^−6^	positive
4544	CYB5R2	0.61	3.49 × 10^−6^	positive	0.68	6.85 × 10^−8^	positive
2227	FLOT1	0.6	7.35 × 10^−6^	positive	0.3	3.84 × 10^−2^	-
2823	GOT1	0.56	1.80 × 10^−5^	positive	0.51	1.55 × 10^−4^	positive
2923	PRKACG	0.56	1.34 × 10^−4^	positive	0.68	5.98 × 10^−7^	positive
632	ARRDC5	0.55	7.40 × 10^−5^	positive	0.67	2.93 × 10^−7^	positive
6391	DPP3	0.54	4.40 × 10^−5^	positive	0.28	5.02 × 10^−2^	-
2997	STOM	0.54	4.85 × 10^−5^	positive	0.46	7.69 × 10^−4^	-
4198	UGP2	0.54	3.44 × 10^−5^	positive	0.37	5.79 × 10^−3^	-
6619	TSPAN16	0.53	2.09 × 10^−4^	positive	0.73	1.64 × 10^−8^	positive
3116	HSPA4	0.52	6.24 × 10^−5^	positive	0.4	2.70 × 10^−3^	-
4301	PYCRL	0.52	2.58 × 10^−4^	positive	0.29	5.51 × 10^−2^	-
1079	TCEB1	0.52	7.86 × 10^−5^	positive	0.56	1.68 × 10^−5^	positive
4332	ACSBG2	0.51	1.31 × 10^−4^	positive	0.59	8.01 × 10^−6^	positive
4838	CAND1	0.51	9.96 × 10^−5^	positive	0.4	3.24 × 10^−3^	-
2426	APCS	0.5	1.27 × 10^−4^	positive	0.62	7.41 × 10^−7^	positive
3976	FHL1	0.49	7.70 × 10^−4^	-	0.61	1.34 × 10^−5^	positive
5742	MYADM	0.49	4.10 × 10^−4^	-	0.55	5.50 × 10^−5^	positive
3025	TPP2	0.48	3.03 × 10^−4^	-	0.57	1.13 × 10^−5^	positive
3972	CUL3	0.47	3.59 × 10^−4^	-	0.54	2.72 × 10^−5^	positive
4179	SYPL1	0.47	5.68 × 10^−4^	-	0.54	4.37 × 10^−5^	positive
4655	GLB1L	0.46	1.67 × 10^−3^	-	0.61	1.06 × 10^−5^	positive
6705	RUVBL1	0.45	8.14 × 10^−4^	-	0.68	2.41 × 10^−8^	positive
5910	DPCD	0.43	1.28 × 10^−3^	-	0.54	2.94 × 10^−5^	positive
6313	FARSB	0.43	1.94 × 10^−3^	-	0.54	5.55 × 10^−5^	positive
2616	UCHL1	0.43	5.71 × 10^−3^	-	0.51	8.25 × 10^−4^	positive
1810	ACOT7	0.41	9.95 × 10^−3^	-	0.51	9.30 × 10^−4^	positive
6860	CFAP20	0.4	1.00 × 10^−2^	-	0.51	8.26 × 10^−4^	positive
5950	TEX101	0.4	7.97 × 10^−3^	-	0.53	2.43 × 10^−4^	positive
1189	TSN	0.39	6.80 × 10^−3^	-	0.56	3.21 × 10^−5^	positive
6190	C12orf10	0.37	1.04 × 10^−2^	-	0.5	3.79 × 10^−4^	positive
2535	PGK2	0.37	5.69 × 10^−3^	-	0.6	1.75 × 10^−6^	positive
6698	RUVBL2	0.36	7.23 × 10^−3^	-	0.53	3.91 × 10^−5^	positive
2721	ACE	0.34	1.32 × 10^−2^	-	0.59	3.06 × 10^−6^	positive
436	EFR3A	0.34	2.81 × 10^−2^	-	0.53	3.71 × 10^−4^	positive
6708	VDAC3	0.34	1.20 × 10^−2^	-	0.52	7.06 × 10^−5^	positive
5290	SLC2A14	0.33	1.81 × 10^−2^	-	0.5	1.49 × 10^−4^	positive
2690	ACADM	0.31	2.80 × 10^−2^	-	0.51	1.79 × 10^−4^	positive
3883	TRAP1	0.29	6.17 × 10^−2^	-	0.52	4.47 × 10^−4^	positive
3502	AQP5	0.24	9.10 × 10^−2^	-	0.58	8.00 × 10^−6^	positive
6150	ACAD9	0.23	1.49 × 10^−1^	-	0.5	7.45 × 10^−4^	positive

Spearman correlation analysis was performed. r ≤ −0.5 was considered as negatively correlated. r ≥ 0.5 was considered as positively correlated. Unpaired two-tailed *t*-test was performed. *p*-value < 0.05 was considered as statistically significant.

## Data Availability

Data are available via ProteomeXchange with the identifier PXD039703.

## Supplementary Materials

The following supporting information can be downloaded at: https://www.mdpi.com/article/10.3390/cells12071017/s1, Table S1: Detailed *t*-test results of proteins comparing subfertile men vs. normozoospermic controls (AN vs. N), asthenozoospermic men vs. normozoospermic controls (A vs. N), and oligoasthenozoospermic men vs. normozoospermic controls (OA vs. N) as determined by LC-MS/MS.; Table S2: Detailed listing of upset plot results in Figure 2B.; Table S3: Detailed results of correlation analysis of all included proteins as determined by LC-MS/MS with the basic semen parameters motility and count.; Table S4: Detailed results of ROC curve analysis of all determined potential biomarker combinations for subfertility in general (AN vs. N), asthenozoospermia (A vs. N), and oligoasthenozoospermia (OA vs. N). Data were presented as the mean of the three-fold cross-validation area under the curve value (Mean CV AUC), the area under the curve (AUC) value of the full data set, the lower and upper confidence interval (CI) with 95% confidence level, and the *p*-value, respectively.; Table S5: More detailed information of participants sperm motility.
