# Supplementary material for: Proteomic Landscape of Human Sperm in Patients with Different Spermatogenic Impairments
Source: Cells. 2023 Mar 26;12(7):1017. doi: 10.3390/cells12071017 (PMC10093380; doi:10.3390/cells12071017)
Supplement: Supplementary file 1 [file cells-12-01017-s001.zip › Supplementary Table S2.pdf]

Supplementary Table S2: Detailed listing of upset plot results in Figure 2B.

Intersection	Size	Proteins
**Only in A vs. N lower**	72	AFM, DBI, MYO1C, DOPEY2, GPR64, MYO1D, SELENBP1, ESPN, SERPINA3, ATP8A1, MGAM, SYTL1, GALE, BASP1, DPP3, SLC44A4, GDI2, FBP1, CD9, GDI1, AZGP1, ANKFY1, GNB1, GLIPR2, SRC, ATP9A, GSS, GNAI2, MON2, IDH1, VAT1, DNAJC5, TSPAN6, CAMK1, MPI, CLIC4, CSTB, NAPA, EZR, ATP6V0A1, CNP, ATP6V1B2, LTA4H, CAP1, QDPR, RAP1A, RAB10, GSR, RAB27A, SYT7, EFHD2, TXNDC17, WDR1, PGD, SERPINB6, ACTN4, GNAS, RAC1, RAB7A, AHCY, MDH1, PARK7, RAB8A, SCFD1, ATP6V1H, RAB5C, RAB18, APEH, TMEM30A, PSMD13, PYCRL, EIF4A1
**Only in A vs. N higher**	53	SAR1A, SEP7, HMOX2, GAPDHS, GAA, TMED4, ENKUR, HSPA9, SCCPDH, PLA1A, HSPD1, LTF, ECH1, MLF1, ATP5O, NFS1, DLD, HADHB, AK8, ACAT1, RPL22, SDHA, FAM209A, CYC1, CABYR, CABYR (iso3), CTSF, CLU, ACO2, SSBP1, OGDH, GK2, ELSPBP1, CAPZA3, COX7C, CPD, NUP210L, CHID1, APOE, H2AFX, HSPE1, SLPI, LRRC37B, CSNK2A1;CSNK2A3, GPC4, PLOD3, DNAH2, IZUMO4, HIST1H1T, SPESP1, CAMP, MMP2, APOA1
**AN vs. N higher & A vs. N higher**	33	P4HB, GANAB, CALR, CDH1, TUBA1A, CPQ, CTSH, HEXA, PPIB, DNAJC3, SIL1, SCPEP1, SEMG1, SEMG2, COL18A1, CPZ, SERPINA5, LAMC1, NEU1, LAMA5, MAMDC2, FN1, GLA, MATN2, SDF4, MXRA5, LAMB2, VWA1, NUCB2, LPL, PLOD1, HSPA13, APOA4
**AN vs. N lower & A vs. N lower**	22	HP, HPX, SERPING1, AHSG, APOH, TTR, PLS3, CYB5R2, ALDH1A1, LCP1, IMPA1, GOT1, APCS, GPI, FLOT1, SYPL1, PGAM2, MYADM, TKFC, ACTG1, HSPA4, CAND1
**Only in AN vs. N lower**	8	STOM, PPP3CC, FHL1, TEX101, PRKACG, TSPAN16, RPS27A, CCT7
**Only in OA vs. N lower**	6	CFAP20, GLB1L, ACADM, ACOT7, TRAP1, RUVBL1
**Only in OA vs. N higher**	1	SRP72
**AN vs. N lower & OA vs. N lower**	1	HDDC2
**Only in AN vs. N higher**	1	CANX
**AN vs. N lower & A vs. N lower & OA vs. N lower**	1	UCHL1
