# Supplementary material for: Proteomic Landscape of Human Sperm in Patients with Different Spermatogenic Impairments
Source: Cells. 2023 Mar 26;12(7):1017. doi: 10.3390/cells12071017 (PMC10093380; doi:10.3390/cells12071017)
Supplement: Supplementary file 1 [file cells-12-01017-s001.zip › Supplementary Table S5.pdf]

Supplementary Table S5: More detailed information of participants sperm motility.

Parameters	N (n=31)	AN (n=22)	OA (n=9)	A (n=13)	N vs. AN	N vs. OA	N vs. A
Total motility (PR + NP, %)	60.2 ± 9.8	50.6 ± 16.0	41.9 ± 12.3	56.7 ± 15.8	*	n.s.	**
Progressive motile (PR, %)	52.0 ± 10.6	12.3 ± 9.0	14.9 ± 11.3	10.5 ± 7.0	***	***	***
Non-progressive motile (NP, %)	7.9 ± 7.0	38.3 ± 20.7	27.0 ± 22.1	46.2 ± 16.2	***	*	***
Immotile (IM, %)	39.8 ± 9.8	49.1 ± 16.2	58.1 ± 12.3	42.8 ± 15.9	*	**	n.s.

- PR = progressive, NP = non-progressive, IM = immotile.
- Data were presented as mean ± standard deviation.
- Unpaired two-tailed t-test was performed.
- *p*-value < 0.05 was considered as statistically significant.
- *** p<0.001; ** p<0.01; * p<0.05; n.s. = not significant.
